# Effects of Multicomponent Injury Prevention Programs on Children and Adolescents’ Fundamental Movement Skills: A Systematic Review With Meta-Analyses

**DOI:** 10.1177/08901171221146434

**Published:** 2022-12-17

**Authors:** John A. Jimenez-Garcia, Matthew B. Miller, Richard G. DeMont

**Affiliations:** 1 Department of Health, Kinesiology, and Applied Physiology, 5618 Concordia University, Montreal, QC, Canada; 2 School of Kinesiology, 8689 Acadia University, Wolfville, NS, Canada

**Keywords:** injury prevention, physical activity, fundamental movement skills, youth, physical literacy

## Abstract

**Objective:**

Fundamental movement skills (FMS) are essential to participate in physical activity. Understanding the effects of multicomponent injury prevention programs (MIPP) on FMS may help promote safe physical activity. Our objective was to synthesize the evidence on the effects of MIPP on biomechanical outcomes and neuromuscular performance measured on children and adolescents while performing FMS.

**Data Source:**

We searched PubMed, SPORTDiscus, Web of Science, and SCOPUS.

**Study Inclusion and Exclusion Criteria:**

We included peer-reviewed randomized controlled trials, published in English, that analyzed the effects of MIPP on biomechanics and neuromuscular performance of FMS in participants under 18 years of age.

**Data Extraction:**

Two reviewers screened the articles, assessed the quality of the evidence using the Physiotherapy Evidence Database (PEDro) scale, and synthesized the data.

**Data Synthesis:**

We conducted meta-analyses and reported the characteristics, outcomes, and risk of bias of studies.

**Results:**

We included 27 articles that reported data from 1,427 participants. Positive effects on FMS were reported in 23 of the 27 included articles. Vertical Jump, running speed, acceleration, and dynamic balance presented positive-significant pooled effect sizes. Dribbling and horizontal jump presented non-significant pooled effect sizes.

**Conclusion:**

MIPP can positively affect FMS in children and adolescents in sports-related settings. Lack of participant compliance and implementation fidelity may affect MIPP effectiveness. Including MIPP in physical literacy interventions, physical education classes, and organized physical activity may lead to functional adaptations that help promote safe physical activity.

## Introduction

High rates of physical inactivity are associated with increased weight status and poor health- and skill-related fitness.^1^ The residual effects of physical inactivity during childhood and adolescence can lead to preventable chronic conditions, such as depression and metabolic and cardiovascular diseases, which increase the risk of all-cause mortality.^1,2^ Globally, more than 80% of 11–17-year-old individuals did not meet the recommended daily physical activity levels, and over 340 million of 5–19-year-old individuals were overweight or obese in 2016.^1^ The prevalence of overweight and obesity among children and adolescents increased from 4% in 1975 to 18% in 2016.^3^

Physical activity promotion efforts aim to decrease pediatric physical inactivity and sedentary behaviour; for instance, the physical literacy model promotes lifelong physical activity by targeting affective (eg, confidence, motivation), cognitive (eg, knowledge, understanding), physical (eg, movement competence), and behavioral (eg, sedentary behavior) factors.^4^ Promoting physical activity is a global objective, yet epidemiological data and injury aetiology models suggest that regular physical activity is associated with an increased risk of musculoskeletal injuries.^5,6^ Musculoskeletal injuries may lead to physical inactivity and chronic musculoskeletal conditions, which further hinder participation in physical activity.^7^ Musculoskeletal injuries have direct economic costs from evaluation, treatment, and rehabilitation and indirect costs when parents are required to attend to an injured child.^8^ Physical activity-related injuries are one of the most important threats to school-aged children and adolescents, and sports and leisure activities were associated with at least 39% of fractures in these populations.^9,10^ The injury risk associated with physical activity reveals the necessity of injury prevention efforts to promote safe physical activity.^11^

Multicomponent injury prevention programs (MIPP) were developed to reduce injury risk and enhance health- and skill-related fitness.^12^ We used MIPP as an umbrella term to generalize standardized injury prevention programs and multicomponent neuromuscular training. When implementing MIPP, researchers and practitioners provide feedback on the exercise technique targeting at least three of the following components: strength, plyometrics, agility, balance, and flexibility.^7^ Systematic frameworks in sports-related settings typically include MIPP; however, physical literacy interventions, physical education curricula, and organized physical activity often omit injury prevention strategies.^13,14^

Fundamental movement skills (FMS) are commonly used in MIPP and play a significant role in physical literacy interventions, physical education, and organized physical activity.^4,15^ FMS are the foundation of specialized movement skills and are classified as locomotion skills (eg, running, jumping), balance skills (eg, static and dynamic balance), and object control skills (eg, kicking, dribbling).^16^ Individuals who do not address FMS deficiencies early in life may be unmotivated and lack the skill to engage in lifelong physical activity and sports, and may also be at an increased risk of musculoskeletal injury.^2,17^ Targeted FMS are used in movement competence and physical literacy assessments as well as injury prevention screening tools.^14^ Previous research proposed a connection between physical literacy and MIPP through the assessment of FMS.^14^

Public health strategies and physical education curricula may benefit from incorporating improved knowledge of the effects of MIPP on FMS in children and adolescents.^18^ MIPP can incorporate developmentally appropriate exercises to improve physical fitness and may lead to engagement in PA through increased confidence and motivation.^2,4^ Other systematic reviews and meta-analyses focused on the effects of MIPP on neuromuscular performance and biomechanical outcomes in children and adolescents,^15,19,20^ but these studies are missing a specific focus on the effects of MIPP on FMS. Our objective was to synthesize the evidence on the effects of MIPP on biomechanical outcomes and neuromuscular performance measured on children and adolescents while performing FMS.

## Methods

We used the Preferred Reporting Items for Systematic Reviews and Meta-Analyses (PRISMA) statement to report this systematic review with meta-analysis.^21^

### Data Sources

We systematically searched PubMed [Medline], SPORTDiscus, Web of Science, and SCOPUS from their inception until July 1st, 2022. We developed a search strategy using the PICOS (Population, Intervention, Comparison, Outcome, Study design) approach and a combination of search terms, synonyms, truncation, and Boolean conjunctions. We then performed citation tracking of key articles, review articles, and authors’ bibliographies to find relevant studies not identified using the search strategy. Search term combinations can be found in the Supplementary Content.

### Inclusion and Exclusion Criteria

We included studies that: (1) included injury-free participants, younger than 18 years of age; (2) implemented at least one intervention incorporating a MIPP; without limits on frequency or duration; (3) used a control group performing either a standard training/warm-up program, sham intervention, or no-treatment; (4) investigated at least one biomechanical outcome and/or neuromuscular performance measured on any FMS; (5) were designed as either a randomized controlled trial (RCT) or a cluster-RCT.

We excluded studies that: (1) included participants who were currently injured or live with a systemic or neurological disease or disability; (2) used an intervention without an injury prevention focus; (3) used a control group performing another exercise-based injury prevention strategy outside their common training routine; (4) did not investigate outcomes measured on FMS; (5) were unavailable or not published in English, and the publication type was an abstract or presentation.

### Data Extraction

Two researchers independently screened titles and abstracts. We removed duplicates and obtained full texts if at least one researcher indicated that the article should be included. Then, the researchers screened full texts and made final inclusion/exclusion decisions. In case of disagreement between the researchers, we consulted a third researcher to achieve consensus to make inclusion/exclusion decisions. One researcher extracted all data, and the second researcher did random data-checks as part of the quality control of the study. We used Rayyan QCRI to manage the search,^22^ Zotero (Corporation for Digital Scholarship, 2020) to manage the references, and a spreadsheet to record the data and decisions. We extracted relevant study information, including authors, year, sample characteristics, outcomes, and intervention and control characteristics. We estimated the level of agreement between researchers in the data extraction process using the Intraclass Correlation coefficient (ICC).

### Risk of Bias Assessment of Individual Studies

Two researchers used the Physiotherapy Evidence Database (PEDro) scale to assess the quality of evidence of included articles independently. The PEDro scale has validity and reliability evidence to assess the quality of RCTs.^23^ Although the PEDro scale uses 11 dichotomous criteria (Yes or No), criterion 1 is not included in the total PEDro score because it pertains to external validity.^23^ Lower values in the PEDro scale indicate potential bias, while higher values in the scale are indicators of high methodological quality.^23^ The researchers were not blinded to the relevant content of the studies (eg, authors’ names). In case of disagreement between the researchers, we consulted a third researcher to achieve consensus on PEDro scores. We estimated the level of agreement between researchers in the quality assessment using the ICC. We did not exclude any study based on the risk of bias assessment.

### Synthesis of Results

#### Narrative synthesis

We synthesized the characteristics of the participants, interventions, and outcomes. We investigated outcomes related to the three FMS categories: locomotion, balance, and object manipulation skills.^16^ The main outcomes were: (1) biomechanical characteristics (eg, joint angles and moments, postural stability) measured on any FMS, and (2) neuromuscular performance (eg, jump height, running speed) measured on any FMS.

We extracted pre- and post-intervention means and standard deviations (SD) from each outcome. If authors reported more than one outcome on the same FMS category in individual studies, we used the following criteria for statistical analyses: (1) If two outcomes were similar (eg, vertical jump with arms and without arms), we used the outcome showing the smaller effect to have a conservative estimate. (2) If three or more outcomes were similar (eg, three reaching directions for Y-balance test without composite score), we used the outcome showing the medium effect.^15^ When data were only available in figures, we extracted the data using an open-source software (Plot Digitizer, http://plotdigitizer.sourceforge.net/).^24^

#### Meta-analyses

We used R 4.0.3 (https://www.r-project.org/) and the package meta to conduct all statistical analyses.^25^ We used an inverse-variance with random-effects model assuming that included studies were methodologically different.^26^ We used the DerSimonian and Laird estimator to pool effect sizes and estimate between-study-variance (*τ*^*2*^). We performed meta-analyses when five or more studies reported the same outcomes to achieve reasonable power for a random-effects model.^27^

We estimated bias-adjusted standardized mean differences (Hedge’s g) of the change scores with 95% confidence intervals (CI) and an alpha level of .05. We symbolized effects in favor of the intervention group with a plus (+) sign and created forest plots with 95% CI to graphically summarize the meta-analyses. We estimated and reported statistical heterogeneity using Cochrane Q and quantified it using *I*^*2*^ statistic; 25%, 50%, and 75% reflect low, moderate, and high heterogeneity, respectively.^28^ To assess the risk of a potential publication bias, we created funnel plots and performed Egger’s regression tests with a .05 alpha level. Funnel plots can be found in the Supplementary Content.

## Results

### Search Results

**Figure 1.**
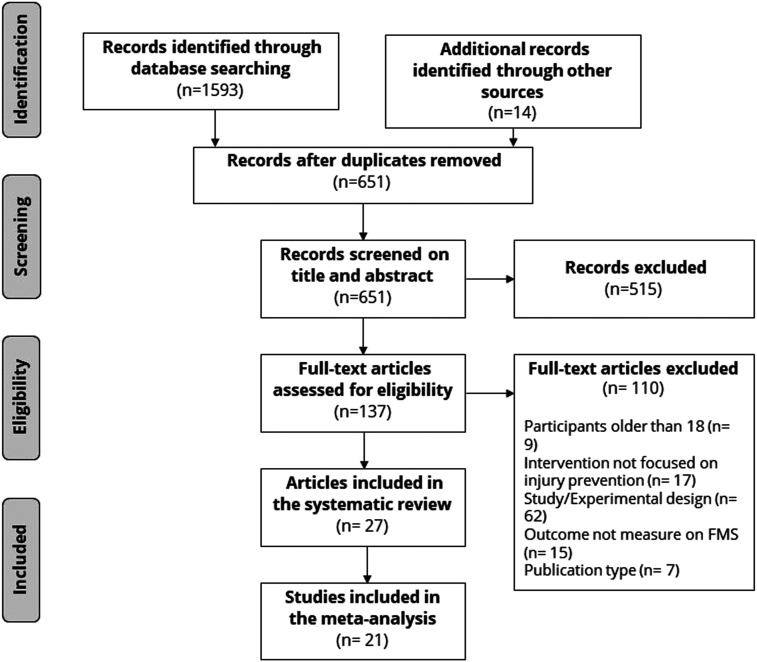
The preferred reporting items for systematic reviews with meta-analysis (PRISMA) flowchart.

We found 28,427 articles (Table 1) using the search strategy. We identified 1593 potentially relevant articles through title screening and 14 articles using other resources. After removing duplicates and screening titles, abstracts, and full texts, we used 27 articles (Figure 1), representing 26 studies. The agreement between the researchers in the data extraction process was excellent, ICC = .953 95% CI [.914, .992].

**Table 1.** Records Identified by Search Strategy and Title Screening.

Database	Records identified using the search strategy	Records identify through title screening
PubMed	8395	443
Scopus	10851	494
Web of science	6245	371
SPORTDiscus	2936	285
Total	28427	1593

### Risk of Bias of Individual Studies

We reported PEDro scores for each study in Table 2 and PEDro detailed results in the Supplementary Content. PEDro scores ranged from four to eight. Nine articles obtained scores above or equal to seven in the PEDro scale,^18,29-36^ and 18 articles scored under seven in the PEDro scale.^37-54^ The agreement between the researchers when using the PEDro scale was excellent, ICC = .941 95% CI: [.906, 0.976].

**Table 2.** Summary of Studies.

Study; Publication Date; Country	Design	Participants (n); Age (years)^a^; Sex (% Female)	Intervention	Duration of intervention; frequency; duration of session	FMS-related outcomes	PEDro score
Kilding et al.; 2008; New Zealand	RCT	24 soccer players; INT: 12; CON: 12; age INT and CON 10.4 (1.4); 0%	INT1: FIFA 11 (modified); CON: No intervention	6 weeks; 5 times per week; 20 min	Countermovement jump; 20 m sprint	6
Steffen et al.; 2008; Norway	RCT	31 soccer players; INT: 17; CON: 14; age INT and CON 17.1 (0.8); 100%	INT: FIFA 11; CON: No intervention	10 weeks; 3 times per week; 15 min	Countermovement jump; 40 m sprint; Slalom dribbling test; Shooting distance	6
Lim et al.; 2009; Korea	RCT	22 basketball players; INT: 11, age 16.2 (1.2); CON: 11, age 16.1 (1.0); 100%	INT: PEP (modified); CON: No intervention	8 weeks; Frequency NA; 20 min	Countermovement jump	6
DiStefano et al.; 2010; United States	Cluster-RCT	65 soccer players; INT1: 19, age 10 (1); INT2: 22, age 10 (1); CON: 24, age 10 (1); 43%	INT1: Progressive pediatric ACL IPP; INT2: Traditional ACL IPP; Control: No intervention	9 weeks; 3 times per week; 12–14 min	Countermovement jump; Time-to-stabilization	6
Vescovi & VanHeest; 2010; United States	RCT	31 soccer players; INT: 15, age 15.7 (1.2); CON: 16, age 16.8 (0.4); 100%	INT: PEP; CON: No intervention	12 weeks; 3 times per week; 15–20 min	9.1 m, 18.3 m, 27.4 m, and 36.6 m Sprint; Countermovement jump	5
Reis et al.; 2013; Portugal	RCT	36 futsal players; INT: 18; CON: 18; age INT and CON 17.3 (0.7); 0%	INT: FIFA 11+; CON: No intervention	12 weeks; 2 times per week; 15–20 min	5 m and 30 m sprint; Slalom dribbling test; Squat jump; Countermovement jump; Single-leg flamingo test	5
Brown et al.; 2014; United States	RCT	23 athletes - teams that require dynamic landings; INT1: 10, age 14.1 (1.2); CON: 13, age 14.7 (2.6); 100%	INT1: Progressive NMT program; CON: No intervention	6 weeks; 3 times per week; 60 min	Unilateral landing after a Standing long jump; Bilateral landing after a forward jump	5
Zech et al.; 2014; Germany	RCT	30 field hockey players; INT: 15, age 15.7 (3.9); CON: 15, age 14.1 (1.4); 0%	INT: Progressive NMT program; CON: No intervention	10 weeks; 2 times per week; 20 min	Star excursion balance test; BESS; Time-to-stabilization; Single-leg stance (center of pressure)	7
Root et al.; 2015; United States	RCT	89 students - members of school sport teams; INT1: 27, age 13 (2); CON1: 32, age 13 (1); CON2: 30, age 13 (2); 33%	INT1: ACL IPP; CON1: Static warm-up program; CON2: Dynamic warm-up program	1 session; 10–12 min	LESS; Countermovement jump; Standing long jump; Shuttle run (30 m down-and-back twice)	8
Rössler et al.; 2016; Switzerland	Cluster-RCT	122 soccer players; INT: 56, age 10.0 (1.8); CON: 66, age 10.1 (1.6); 0%	INT: FIFA 11+ Kids; CON: Sham treatment	10 weeks; 2 times per week; 15 min	Single-leg stance (center of pressure); Y-balance test; Countermovement jump; Standing long jump; 20 m Sprint; Slalom dribbling test; Wall-volley test	7
Ayala et al.; 2017; Spain	RCT	41 soccer players; INT1: 10; CON1: 11; INT2: 10; CON2: 10; age INT1, INT2, CON1 and CON2 16.8 (0.7); 0%	INT1: FIFA 11+; CON1: No intervention; INT2: Harmoknee warm-up program; CON2: No intervention	4 weeks; 3 times per week; 20 min	Y-balance test; Single-leg hop for distance; Triple-hop for distance; 10 m and 20 m Sprint	7
Ondra et al.; 2017; Czech Republic	RCT	21 basketball players; INT: 10, age 17.3 (1.3); CON: 11, age 16.5 (1.8); 0%	INT: Propioceptive and NMT program; CON: No intervention	20 weeks; 3 times per week; 20 min	Single-leg stance (center of pressure)	6
Akbari et al.; 2018 and 2019; Iran	RCT	24 soccer players; INT: 12; CON: 12; age INT and CON 16.79 (1.18); 0%	INT: FIFA 11+; CON: No intervention	8 weeks; 3 times per week; 20–25 min	Vertical jump; LESS	6
De Ste. Croix et al.; 2018; United Kingdom	Cluster-RCT	125 soccer players; INT: 71, age 13.1 (1.7); CON: 54, age 12.8 (1.6); 100%	INT: Progressive multicomponent robustness training program; CON: No intervention	16 weeks; 3 times per week (1 coach-led warm-up + 2 player-led sessions); 20 min	Submaximal hopping protocol (20 hops to test leg stiffness); Single-leg countermovement jump (vertical stop-jump task)	7
Gatterer et al.; 2018; Austria	RCT	16 soccer players; INT: 8; CON: 8; age INT and CON 10; 0%	INT: FIFA 11+; CON: No intervention	5 weeks; 2 times per week; 30 min	Standing long jump; Stability	6
Pomares-Noguera et al.; 2018; Spain	RCT	23 soccer players; INT: 13; CON: 10; age INT and CON 11.8 (0.3); 0%	INT: FIFA 11+ Kids; CON: No intervention	4 weeks; 2 times per week; 15–20 min	Y-balance test; 20 m sprint; Countermovement jump; Standing long jump; Slalom dribbling test; Wall-volley test	7
Taylor et al.; 2018; United States	Cluster-RCT	97 basketball and soccer players; INT: 48, age 15.4 (1.0); CON: 49, age 15.7 (1.6); 100%	INT: Warm-up-based ACL IPP; CON: No intervention	6 weeks; 2–3 times per week; 20–25 min	Countermovement jump; Single-leg hop test; Standing long jump; Single-leg hop (distance); Lateral jump; Triple-hop test; Single-leg lateral jump	7
Zarei et al.; 2018; Iran	Cluster-RCT	66 soccer players; INT: 34, age 15.03 (0.7); CON: 32, age 15.22 (0.6); 0%	INT: FIFA 11+; CON: No intervention	30 weeks; 2 times per week; 20-25 min	Dribbling sprint test; 9.1 m and 36.6 m sprint; Vertical jump	5
Zarei et al.; 2018; Iran	RCT	42 soccer players; INT: 19, age 11.93 (1.91); CON: 23, age 12.16 (1.13); 0%	INT: FIFA 11+ Kids; CON: No intervention	10 weeks; 3 times per week; 20 min	Slalom dribbling test; Standing long jump; Triple-hop for distance; Y-balance test; 20 yard and 40 yard sprint	4
McKenzie et al.; 2019; New Zealand	Cluster-RCT	81 netball players; INT: 45, age 13.33 (0.34); CON: 36, age 13.25 (0.50); 100%	INT: Netball Smart Dynamic Warm-up (NDW); CON: No intervention	7 weeks; 3 times per week; 15–20 min	Y-balance test; 20 m sprint; Standing long jump; Countermovement jump; Time-to-stabilization	5
Pardos-Mainier et al.; 2019; Spain	RCT	32 soccer players; INT: 15, age 12.5 (0.4); CON: 17, age 13.1 (0.3); 100%	INT: FIFA 11+; CON: No intervention	10 weeks; 2 times per week; 15–20 min	Standing long jump; Single-leg hop test; Countermovement jump; Single-leg countermovement jump; Y-balance test	5
Parsons et al.; 2019; Canada	Cluster-RCT	43 soccer players; INT: 25, age 11.1 (Range 10.1-11.7); CON: 18, age 10.8 (Range 9.5-11.7); 100%	INT: FIFA 11+; CON: No intervention	Indoor soccer season (5 month); 1–3 times per week; 20 min	LESS; Y-balance test; Countermovement jump	7
Trajkovic & Bogataj; 2020; Serbia	RCT	66 volleyball players; INT: 32, age 11.12 (0.68); CON: 34, age 10.96 (0.75); 100%	INT: Progressive NMT program; CON: No intervention	10 weeks; 2 times per week; 30 min	Lateral jumps; Single-leg hop test; 10 m sprint; Countermovement jump; Medicine ball Throw	6
Forrest et al.; 2020; Australia	Cluster-RCT	65 cricket players; INT: 32, age 15.8 (1.0); CON: 33, age 15.4 (1.2); 0%	INT: Progressive cricket IPP; CON: No intervention	8 weeks; 2 times per week; 15 min	SEBT	7
Font-Lladó et al.; 2020; Spain	RCT	190 children; INT 97, age 7.47 (0.27); CON 93, age 7.39 (0.38); 52.63%	INT: Pedagogical Integrative NTM; CON: No Intervention	12 weeks; 2 times per week; 20 min	CAMSA (FMS score)	6
Teixeira et al.; 2021; Brazil	RCT	24 soccer players; INT: 12, age 10.0 (0.6); CON: 12, age 9.75 (0.75); 0%	INT: FIFA 11+ Kids; CON: No intervention	8 weeks; 3 times per week; 30 min	Countermovement jump	5

^a^Data are mean (SD) except where otherwise stated. IPP = injury prevention program; NMT = neuromuscular training; RCT = randomized controlled trial; INT = intervention group; CON = control group; NA = not available; PEP = prevent injuries enhance performance; ACL = anterior cruciate ligament; LESS = Landing Error Scoring System; BESS = Balance Error Scoring System; CAMSA = Canadian Agility and Movement Competence Assessment.

### Characteristics of Included Interventions and Participants

We reported detailed information for each article in Table 2. Included articles involved 1427 participants of which 715 received an intervention and 49.3% were females (*n* = 703). The participants’ median age was 13.72 years, ranging 7.39–17.30. Ten articles reported data on females only,^18,30,31,39,43,44,46,48-50^ 14 articles on males only,^29,32,34,35,37,38,41,42,45,47,51,52^ and three articles on both females and males.^33,40^ Fourteen articles analyzed participants younger than 14 years,^30-34,40-42,44,46,49,52^ and 13 articles analyzed participants older than 14 years.^18,29,35,37-39,43,45,47,48,50,51^

All interventions combined at least three of the following components: strength, flexibility, plyometrics, balance, and agility. Fifty-six percent of the interventions were 6–10 weeks in length, 88.9% of the interventions had 2-3 sessions per week, and 70.37% of the sessions lasted 11–20 min. Twenty-five articles used MIPP as warm-ups and made comparison to regular warm-ups at different sports settings.^18,29-38,40-48,50-54^ Two studies used MIPP as part of a session after warming up and made comparisons to a no-treatment conditions.^39,49^ Fourteen articles reported a version of the Fédération Internationale de Football Association (FIFA) 11+ (ie “11”, FIFA 11+, FIFA 11 + Kids),^29,31,32,34,37,38,41,42,46-48,51,52,54^ six articles reported neuromuscular training,^30,35,39,45,49,53^ three articles reported anterior cruciate ligament (ACL) injury prevention programs,^18,33,40^ three articles reported different versions of standardized IPP (ie, Preventing Injury Enhancing Performance, Netball Dynamic Warm-up, and Harmoknee),^44,46,50^ and one article reported an original IPP or cricket.^36^ From the 27 articles, one article compared the FIFA 11+ and the Harmoknee program against control groups.^29^ Sixteen articles reported data on soccer players,^29-32,34,37,38,40-42,46,48,50-52,54^ three articles on basketball players,^18,43,45^ seven articles on other sports (ie, field hockey, volleyball, futsal, netball, cricket),^33,35,36,39,44,47,49^ and one article on an school-based setting.^53^ Nineteen articles were RCT,^29,32,33,35,38,39,41-43,45-50,52-54^ and eight articles were cluster-RCT.^18,30,31,34,36,40,44,51^

### Outcome Measures

Included articles reported outcomes on all FMS categories (Table 2). Ten articles reported outcomes on locomotion skills,^18,30,33,37-39,42,43,50,54^ three articles on balance skills,^35,36,45^ seven articles on locomotion and balance skills,^29,31,40,41,44,46,49^ two articles on locomotion and object manipulation,^48,51^ and five articles on the three FMS categories.^32,34,47,51,53^ Regarding the type of outcomes, eight articles reported outcomes on neuromuscular performance,^29,32,36,37,42,46,47,50^ seven articles on biomechanics,^18,30,38,39,45,52,54^ ten articles on both neuromuscular performance and biomechanics,^31,33-35,40,41,43,44,48,51^ and two articles on neuromuscular performance and movement competence.^49,53^

### Effects of the Interventions

Twenty-one articles studied the effects of MIPP compared to control groups by using frequentist statistics and determining statistical significance based on P-values. Eight articles reported positive-statistically-significant effects of MIPP in all their outcomes.^37,38,42,45,47,49^ Positive effects were reported for vertical jump performance,^37,38,42,47^ running performance,^42,47^ postural stability,^36,45^ landing technique,^37,38^ dribbling in soccer,^47^ the motor quotient in the Körperkoordinationstest für Kinder (movement competence assessment tool),^49^ and the FMS score in the Canadian Agility and Movement Skill Assessment (CAMSA).^53^

Nine articles reported a combination of positive-statistically-significant and non-statistically-significant effects of MIPP in their outcomes.^33,35,39,40,43,44,46,52^ Significant positive effects were reported for vertical jump performance,^40,44^ running performance,^51^ knee flexion angle,^43^ between knee distance,^43^ maximal knee abduction torque,^43^ postural stability,^35,40,46^ hip flexion angle,^39^ landing technique,^33^ dynamic balance,^44,51^ triple hop test,^52^ impulse peak force,^54^ and maximum impulse force.^54^ Non-statistically-significant effects were reported for vertical jump performance,^33,40,43,46^ horizontal jump performance,^33,44^ running performance,^33,44^ maximal knee internal rotation angle,^43^ postural stability,^35,40,46^ hip adduction,^39^ knee flexion,^39^ knee abduction,^39^ dynamic balance,^35^ center of pressure,^35^ dribbling in soccer,^51^ single-leg hop test,^46^ jump duration time,^54^ maximum power output,^54^ and force development rate.^54^

Four articles reported non-statistically-significant effects of MIPP in all their outcomes.^18,31,48,50^ Non-statistically-significant effects were reported for vertical jump performance,^48,50^ running performance,^48,50^ knee valgus angle,^48^ dribbling in soccer,^48^ hip flexion,^18^ hip adduction,^18^ hip internal rotation,^18^ knee flexion,^18^ knee abduction,^18^ knee internal rotation,^18^ landing technique,^31^ and dynamic balance.^31^

Six articles used magnitude-based inferences to study the effects of the MIPP compared to control groups.^29,30,32,34,41,52^ Magnitude-based inferences are based on uncertainty in the true value of a statistic, which is expressed as confidence limits. The confidence limits are interpreted based on a three-level scale of magnitudes: beneficial, trivial, and harmful; then, an associated likelihood is stated.^55^ Beneficial effects and their likelihoods were reported for vertical jump performance,^29,32,34,51^ running performance,^29,51^ horizontal jump performance,^32,34^ dynamic balance,^29,32,34^ reactive strength index of vertical jump after a drop jump,^34^ dribbling in soccer,^34^ leg stiffness,^29,30^ knee valgus,^30^ and sensory and stability indices.^41^ Trivial effects were reported for single-leg stance,^34^ running performance,^34^ and dribbling in soccer ^32^; conversely, one article reported possible harmful effects for dribbling sprint test in soccer.^51^

### Meta-Analyses

**Figure 2.**
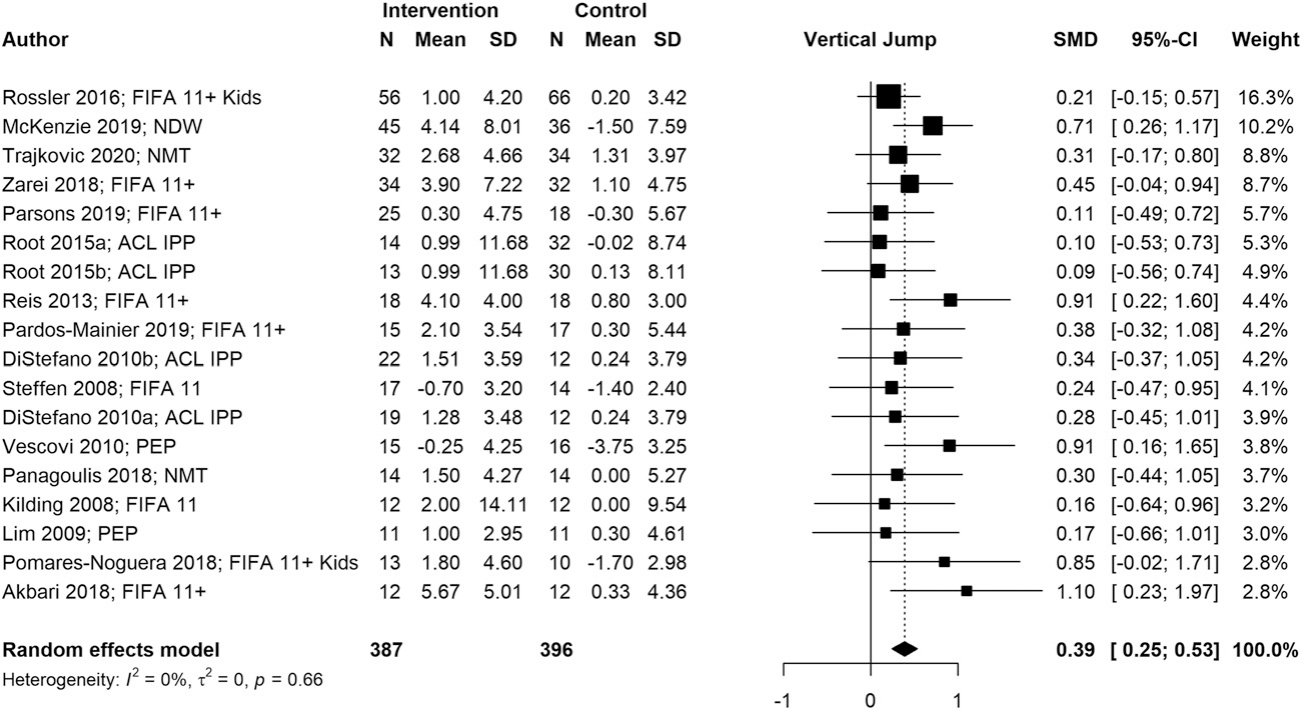
Forest plot and pooled effect size for vertical jump performance. SMD = Standardized mean difference; ACL = Anterior Cruciate Ligament; IPP = injury prevention program; NMT = neuromuscular training; PEP = prevent injuries enhance performance; NWD = Netball Dynamic Warm-up.

**Figure 3.**
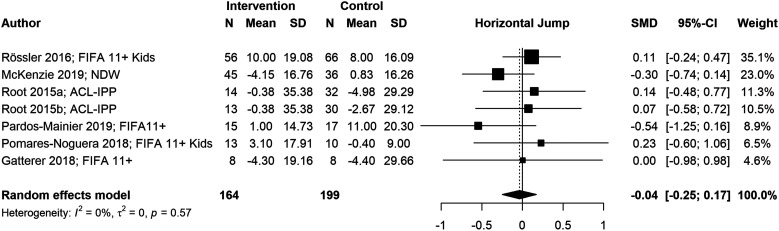
Forest plot and pooled effect size for horizontal jump performance. SMD = Standardized mean difference; ACL = Anterior Cruciate Ligament; IPP = injury prevention program; NWD = Netball Dynamic Warm-up.

**Figure 4.**
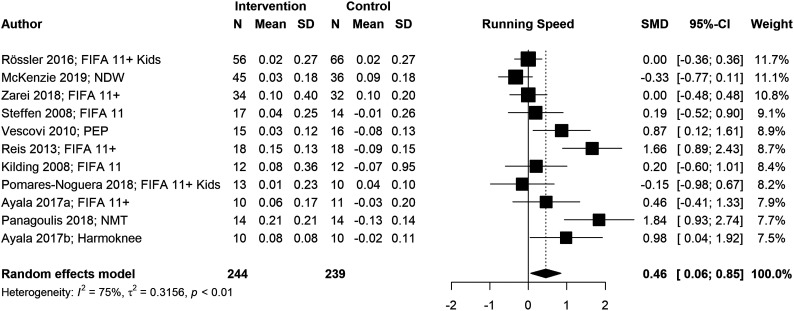
Forest plot and pooled effect size for running speed. SMD = Standardized mean difference; NMT = neuromuscular training; PEP = prevent injuries enhance performance; NWD = Netball Dynamic Warm-up.

**Figure 5.**
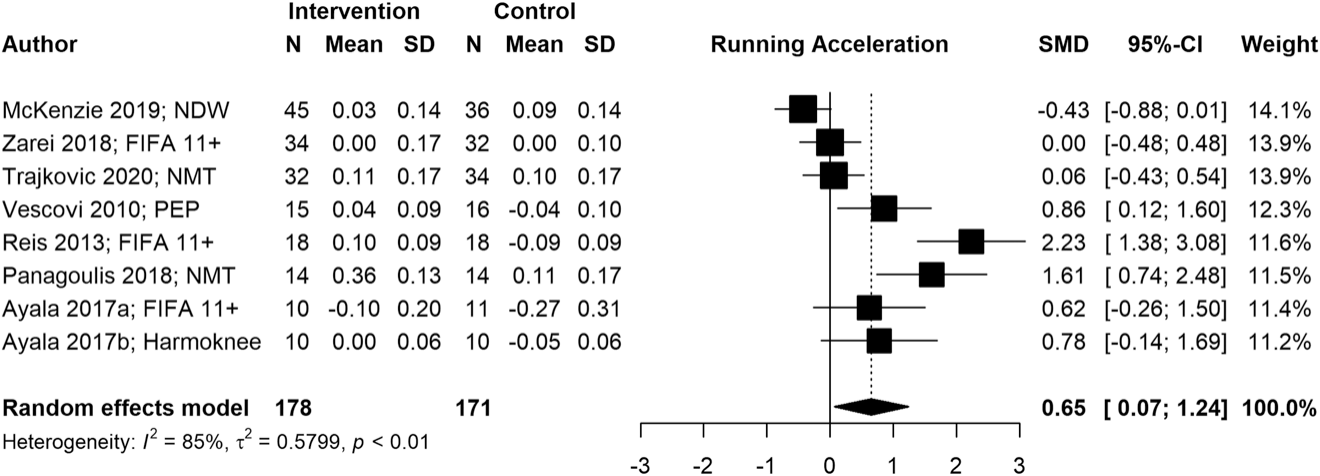
Forest plot and pooled effect size for running acceleration. SMD = Standardized mean difference; NMT = neuromuscular training; PEP = prevent injuries enhance performance; NWD = Netball Dynamic Warm-up.

**Figure 6.**
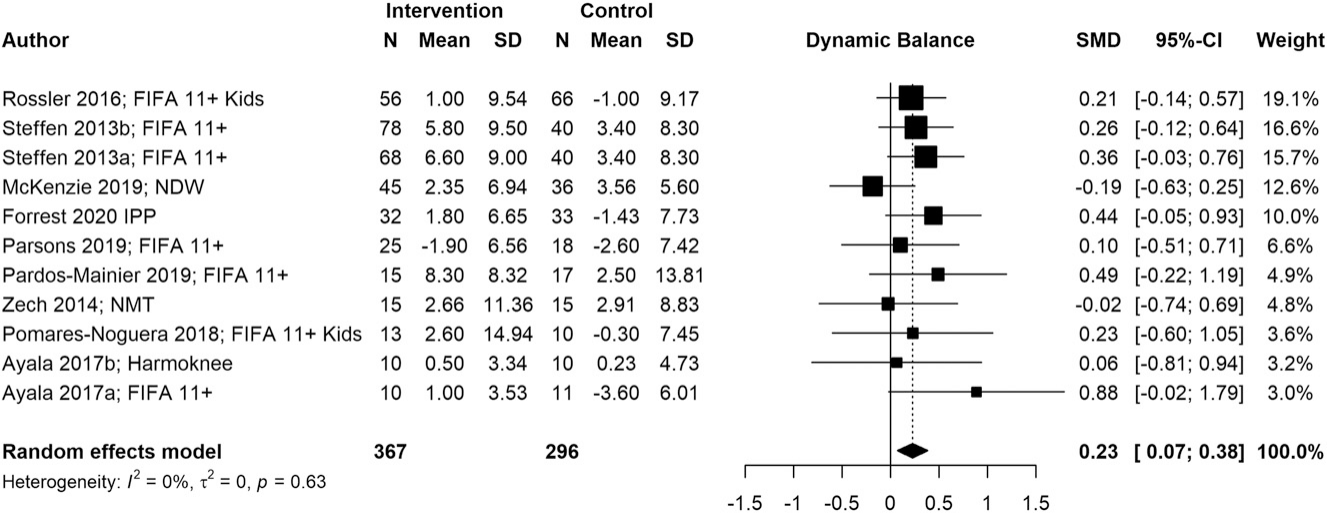
Forest plot and pooled effect size for dynamic balance. SMD = standardized mean difference; NMT = neuromuscular training; NWD = netball dynamic warm-up.

**Figure 7.**
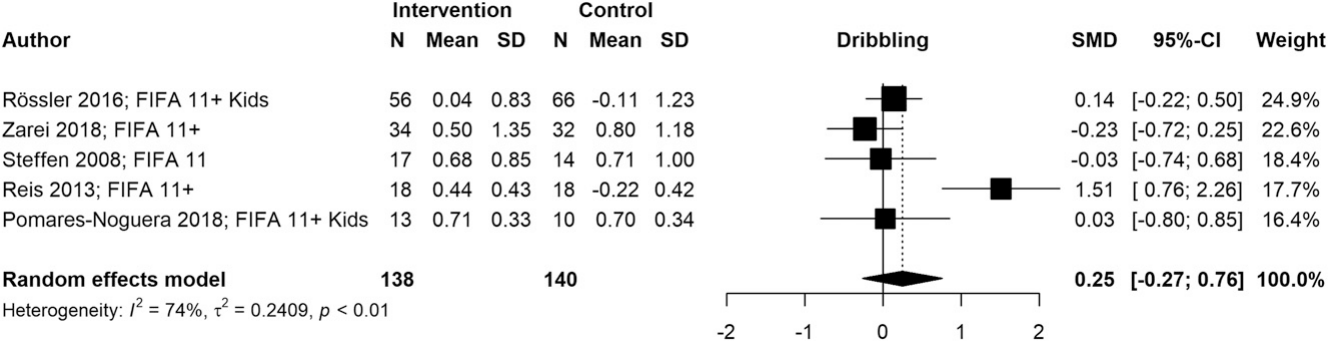
Forest plot and pooled effect size for dribbling. SMD = Standardized mean difference.

We conducted six meta-analyses to estimate pooled effect sizes based on the outcomes that were reported in at least five studies. Meta-analyses for locomotor skills were completed on 738 participants for vertical jump performance (Figure 2), 363 participants for horizontal jump performance (Figure 3), 483 participants for running speed (Figure 4), and 349 participants for running acceleration (Figure 5). Running performance was divided into speed when measured between 10 m and 40 m, and acceleration when measured between 0 m and 10 m. Meta-analyses for balance skills and object manipulation skills were completed on 663 participants for dynamic balance (Figure 6), and 278 participants for dribbling in soccer (Figure 7).

#### Locomotor skills

We observed significant positive pooled effect sizes for vertical jump (g = .39; 95% CI: [.25, .53]; *p* < .001), running speed (g = .46; 95% CI: [.06, .85]; *p* = .024), and running acceleration (g = .65; 95% CI: [.07, 1.24]; *p* = .028). Conversely, horizontal jump presented a non-significant negative pooled effect size (g = −.04; 95% CI: [−.25, .17]; *p* = .724). Vertical jump and horizontal jump presented low heterogeneity (*I*^*2*^ = 0%), indicating that included effect sizes were similar. Running speed (*I*^*2*^ = 75%) and running acceleration (*I*^*2*^ = 84%) presented high heterogeneity, indicating variability among effect sizes of individual studies.

#### Balance skills

We observed a significant positive pooled effect size for dynamic balance (g = .23; 95% CI: [.07, .38]; *p* = .004). The low heterogeneity of the meta-analysis (*I*^*2*^ = .0%) indicated that included effect sizes were similar.

#### Object control skills

We observed a non-significant positive pooled effect size for dribbling (g = .25; 95% CI: [−.27, .76]; *p* = .345). The high heterogeneity of the meta-analysis (*I*^*2*^ = 74%) indicated variability among effect sizes of individual studies.

### Risk of Publication Bias Assessment

Funnel plots and Egger’s regression tests did not suggest a risk of publication bias in vertical jump (*p* = .377), horizontal jump (*p* = .903), dynamic balance (*p* = .655), and dribbling (*p* = .560) measures. Significant Egger’s test confirmed funnel plot asymmetry in running speed (*p* = .023) and running acceleration (*p* = .006) meta-analyses.

## Discussion

The objective of this study was to synthesize the evidence on the effects of MIPP on biomechanical outcomes and neuromuscular performance measured on children and adolescents while performing FMS. Included studies reported that MIPP had a combination of positive-statistically-significant effects and non-statistically-significant effects on the biomechanics and neuromuscular performance of the three categories of FMS: locomotion, balance, and object manipulation skills. At least one positive/beneficial effect was reported in 23 of the 27 included articles. Meta-analyses showed significant positive pooled effects sizes for vertical jump performance, running speed, running acceleration, and dynamic balance, non-significant positive pooled effect size for dribbling, and non-significant negative pooled effect size for horizontal jump performance. Considering the relevance of FMS to promote physical activity in multiple contexts, the implementation of MIPP in physical literacy interventions, physical education classes, and organized physical activity is supported by the positive effects of MIPP in specific FMS and their reported effectiveness in reducing injury rates.^11^

### Multicomponent Injury Prevention Programs

The characteristics of the MIPP studied on this review are similar to the characteristics of the interventions studied in other systematic reviews and meta-analyses focused on the same population.^11,15,20,56^ All MIPP in the included articles, combined at least three components from which strength, plyometrics, and balance components were the most used. Most of the included articles used MIPP 2-3 times per week during 6–10 weeks with 11–20-minute sessions. Twenty-five of the 27 articles used MIPP as a warm-up, which agrees with Lim et al. (2009) and Root et al. (2015) who suggested that MIPP are suitable warm-ups as they can induce acute and beneficial adaptations before an intense session where injuries can occur.^43,57^

Twenty-six of the 27 interventions were implemented in sports-related settings suggesting a potential underuse of MIPP in other contexts. Considering that participating in physical activity increases the risk of injury,^6,19^ MIPP should be implemented in contexts such as physical literacy interventions, physical education classes, and organized physical activity. The potential underuse of MIPP in multiple contexts is unfortunate because meta-analytical data indicates that MIPP can result in an injury reduction of around 46% in sports-related settings.^11^ The preventive effects of MIPP are associated to the modification of multiple risk factors of injury, such as postural control, strength, or flexibility deficits.^35,58^ Although the primary objective of MIPP is to affect modifiable risk factors of injury, other reviews^15,20,56^ and our meta-analyses reported improvements in neuromuscular performance and biomechanics in children and adolescents.

### Effects of Multicomponent Injury Prevention Programs on Fundamental Movement Skills

The effects of MIPP on strength, power, agility, flexibility, and balance may act synergically to mitigate biomechanical risk factors for injury and improve neuromuscular performance in FMS.^7,59^ Neuromuscular adaptations from MIPP result in motor unit coordination, firing, and recruitment, which are essential factors for the quality of the movement.^15,60,61^ These neuromuscular adaptations along with the benefits of strength and plyometric training lead to locomotor skills improvements.^15,60,62^ Plyometric exercises included in MIPP may induce adaptations in muscles’ contractile elements and enhanced efficiency of the stretch-shorten-cycle function, which benefits unilateral and bilateral jumping performance.^37^ The significant positive pooled effect size (g = .39) with narrow CI for vertical jump performance reflect these neuromuscular adaptations. Similarly, improved running performance (g = .46 for running speed and g = .65 for running acceleration) may be the product of enhanced neuromuscular activation (eg, firing frequency of motor units), improved ground contact time, and increased musculotendon unit stiffness.^63^ Leg stiffness improvements contribute to a change in the activation of the musculotendon unit leading to increased pre-activation before ground contact (ie, feed-forward control) and increased co-contraction after ground contact (ie, feedback control), thus promoting enhanced stability upon landing in unilateral and bilateral tasks.^30^

The effectiveness of MIPP on landing mechanics may be the result of core and hip exercises as well as the feedback provided to correct lower-extremity and trunk alignment.^38^ Hip abduction strength contributed to improved control of frontal-plane knee and hip motions during unilateral and bilateral landing tasks.^30,32^ Meta-analytical data indicate that MIPP have the potential for successful modification in high-risk lower limb landing mechanics that lead to decreased lower limb injury rates, indicating that biomechanical adaptations are exercise-dependent.^20,39^ For instance, Brown et al. (2014) reported that landing with increased knee flexion seems dependent on the training modality and the feedback associated with each training component.^39^

Balance exercises included in MIPP may induce task-specific neurological adaptations, suppress muscle stretching reflex excitability during postural tasks, and enhance co-contraction between agonist and antagonist muscles.^52,64^ Enhanced control over center of gravity shifts and automatic postural response patterns are the likely mechanisms that account for the positive pooled effect size (g = .23) with narrow CI observed in the dynamic balance meta-analysis and balance performance in general.^45,65,66^ Although balance was a small component of MIPP, individual studies and our meta-analysis reported positive effects suggesting that intense balance training programs may not be necessary to observe improvements in balance.^40^

Individual studies and our meta-analyses suggest that MIPP have the potential to induce positive effects on specific FMS; however, the success of MIPP is not universal and is influenced by various factors. Participant compliance, implementation fidelity, and adherence issues and the characteristics of the interventions can affect the effectiveness of MIPP.^18,48,50^ Participants with the greatest compliance seem to experience greatest effects on biomechanical outcomes, neuromuscular performance, and injury rate reduction.^57,67^ Parsons et al. (2019) indicated that only the participants who were most adherent to the intervention improved in dynamic balance performance.^31^ Similarly, Steffen et al., (2013) reported a dose-response relationship between the number of sessions/exercises and performance.^67^ Several factors can affect compliance; for instance, Kilding et al. (2008) stated that the intervention was repetitive and caused boredom in the participants, which may affect their willingness to actively participate in it.^42^ Moreover, Vescovi and VanHeest (2010) suggested that stakeholders might be unwilling to include additional exercises to reduce injury risk because it may take too much time from the training/practice session, which directly affects implementation fidelity.^50^

Lack of participant compliance and implementation fidelity are well recognized problems in MIPP, but the literature provides some remedies. Compliance can be enhanced by including group activities and implementing sessions that require little time commitment.^43^ Progressive exercises can also enhance participants’ enjoyment, and an early intervention may help children and adolescent to get used to the routine and protocols of MIPP, resulting in better long-term compliance.^40,57^ Padua et al. (2014) suggested that achieving participant compliance, long-term adoption, implementation fidelity, and sustainability of MIPP require the development of administrative support within the organizations.^68^ Stakeholders must support the use of MIPP to promote widespread dissemination.^40^ Demonstrating an injury rate reduction along with acute improvements in biomechanics and neuromuscular performance in FMS may provide instant gratification to stakeholders that may help enhance compliance.^57^

MIPP characteristics are also addressed by authors to explain their non-positive results. Some MIPP with varying levels of training volume, intensity, progression, and content might have been insufficient to improve performance in specific studies.^18,31,48,50^ Taylor et al. (2018) suggested that MIPP may not provide the appropriate stimulus to modify lower extremity biomechanics within a 6-week period with two sessions per week.^18^ Additionally, Taylor also stated that MIPP often emphasize on double-leg and sagittal plane movements hindering biomechanical adaptations during single-legged and/or frontal plane movements.^18^ Gatterer et al. (2018), McKenzie et al. (2019), and Pardos-Mainier et al. (2019) reported that MIPP often do not include many horizontal jumps, which may explain the non-significant negative pooled effect (g = −.04) in horizontal jump performance.^41,44,46^ The focus and feedback strategy used on MIPP can also affect the effectiveness of the interventions.^7^ Vescovi and VanHeest (2010) suggested that MIPP that heavily focus on reducing landing forces may fail to improve jump performance.^50^ Conversely, Steffen et al. (2008) reported that participants might have developed leg power, but a poor technique may explain the poor jumping performance, suggesting that more feedback was needed.^48^

Dribbling was the only object control skill with the sufficient data to conduct a meta-analysis. Although dribbling was particular to soccer, it provided insights into the effects of MIPP in context-specific tasks. The pooled effect size of dribbling was positive (g = .25) but non-significant; specifically, three studies reported positive effects,^32,34,47^ and two studies reported negative effects.^48,52^ Since dribbling was assessed in a soccer context, the ambiguous results may be due to highly specialized and skilled participants with little room for improvement. Despite these results, developing MIPP that include object control skills is relevant because improved neuromuscular control during these skills may enable participants to process environmental stimuli better and faster, favoring the attentional capacity and movement competence.^15^

### Limitations

This systematic review with meta-analysis has some limitations. Although we defined a priori inclusion and exclusion criteria, we conducted the narrative synthesis and meta-analyses based on substantially different studies regarding the participants, content, characteristics, and outcomes of the interventions. For example, assessments in selected studies were different, and their comparability was affected, which may lead to discrepancies in the results. We investigated outcomes related to all FMS categories; however, included studies did not report on some common FMS (eg, leaping and galloping). We extracted and reported a series of biomechanical and neuromuscular performance outcomes, but we could not conduct meta-analyses for any biomechanical outcome because we required at least five studies reporting the same outcome to achieve reasonable power for a random-effects model.^27^ Running speed, acceleration, and dribbling meta-analyses should be cautiously interpreted due to their high heterogeneity values. Potential publication bias indicates that running speed and acceleration pooled effect sizes may be overestimated despite their significant positive results. Only the first author extracted the data, which increases the probability of human mistakes in the data extraction process; however, a second researcher did random data-checks as part of the quality control of the systematic review.

## Conclusions

MIPP positively influenced specific biomechanical outcomes and neuromuscular performance measured on children and adolescents while performing FMS. Short MIPP that focus on progression and a variety of movement skills should be implemented at the beginning of a session as lengthy interventions can negatively affect participant compliance.^39,42^ Properly designed MIPP must consider training specificity, intensity, and volume to provide enough stimuli to lead to positive biomechanical and neuromuscular performance effects in FMS. Stakeholders’ involvement needs to be prioritized to enhance implementation fidelity. Athletes benefited from MIPP in sports-related settings, so it is plausible that less specialized individuals will also benefit from MIPP; moreover, implementing MIPP in physical literacy interventions, physical education classes, and organized physical activity may help promote safe physical activity as MIPP can reduce injury risk and positively affected neuromuscular performance.

**So What?**

**What is Already Known on This Topic?**

Multicomponent injury prevention programs (MIPP) are used to reduce musculoskeletal injury risk and enhance health- and -skill-related fitness. Fundamental movement skills (FMS) are commonly used in MIPP and play a significant role in physical literacy, physical education, and organized physical activity.

**What Does This Article Add?**

MIPP positively affected specific biomechanical outcomes and neuromuscular performance measured in children and adolescents while performing FMS. Implementing MIPP in physical literacy, physical education, and organized physical activity may help promote safe physical activity as FMS are used and assessed in the physical activity and injury prevention fields.

**What are the Implications for Health Promotion Practitioners or Research?**

The potential functional adaptations and the preventive capacity of MIPP are relevant arguments to convince stakeholders to implement MIPP outside sport-related contexts. Lack of compliance reduces the potential effects of MIPP. Future research should investigate the implementation of MIPP outside sport-related contexts.

## Supplemental Material

Supplemental Material - Effects of Multicomponent Injury Prevention Programs on Children and Adolescents’ Fundamental Movement Skills: A Systematic Review With Meta-Analyses Click here for additional data file. Supplemental Material for Effects of Multicomponent Injury Prevention Programs on Children and Adolescents’ Fundamental Movement Skills: A Systematic Review With Meta-Analyses by John A. Jimenez-Garcia, Matthew B. Miller, and Richard G. DeMont in American Journal of Health Promotion
