# Supplementary material for: Effects of Multicomponent Injury Prevention Programs on Children and Adolescents’ Fundamental Movement Skills: A Systematic Review With Meta-Analyses
Source: Am J Health Promot. 2022 Dec 17;37(5):705–19. doi: 10.1177/08901171221146434 (PMC10580683; doi:10.1177/08901171221146434)

Supplementary Online Content

Effects of Multicomponent Injury Prevention Programs on Children and Adolescents' Fundamental Movement Skills: A Systematic Review with Meta-Analyses

Appendix A. Search Strategy
- Table A1. Search Strategy

Appendix B. Risk of bias Assessment
- Table B1. Physiotherapy Evidence Database (PEDro) scores.

Appendix C. Risk of publication bias plots
- Figure C1. Funnel plot for vertical jump.
- Figure C2. Funnel plot for horizontal jump.
- Figure C3. Funnel plot for dynamic balance.
- Figure C4. Funnel plot for running speed.
- Figure C5. Funnel plot for running acceleration.
- Figure C6. Funnel plot for dribbling.

Appendix A. Search Strategy

Table A1. Search Strategy.

Database	Search Strategy/Phrase
PubMed	(((child[Title/Abstract] OR children[Title/Abstract] OR kid[Title/Abstract] OR kids[Title/Abstract]) OR child[MeSH Major Topic]) OR ((adolescent[Title/Abstract] OR adolescents[Title/Abstract]) OR adolescent[MeSH Major Topic]) OR ((youth[Title/Abstract] OR young[Title/Abstract]) OR youth sports[MeSH Major Topic])) AND (((injury[Title/Abstract] OR injuries[Title/Abstract]) OR athletic injuries[MeSH Major Topic]) AND (prevention[Title/Abstract] OR preventative[Title/Abstract]) OR (program[Title/Abstract] OR programs[Title/Abstract] OR programme[Title/Abstract] OR programmes[Title/Abstract]) OR ((warm up[Title/Abstract] OR warm-up[Title/Abstract]) OR warm up exercise[MeSH Major Topic]) OR (neuromuscular[Title/Abstract] OR neuromuscular training[Title/Abstract] OR integrative neuromuscular training[Title/Abstract])) AND ((athletic performance[MeSH Major Topic]) OR ((balance[Title/Abstract] OR dynamic balance[Title/Abstract] OR static balance[Title/Abstract] OR stability[Title/Abstract]) OR postural balance[MeSH Major Topic]) OR ((biomechanic[Title/Abstract] OR biomechanics[Title/Abstract] OR biomechanical[Title/Abstract]) OR biomechanical phenomena[Mesh:noexp]) OR (jump OR jumps OR jumping) OR (hop OR hops OR hopping) OR (land OR lands OR landing) OR ((jog OR jogs OR jogging OR sprint OR sprints OR sprinting OR run OR runs OR running) OR running[MeSH Major Topic]) OR (cutting tasks) OR (lower extremity[MeSH Major Topic]) OR (locomotion[MeSH Major Topic]) OR (motor skill[MeSH Major Topic]))
Scopus	(TITLE-ABS-KEY ((child*) OR (kid*) OR (adolescent*) OR (youth OR young))) AND (TITLE-ABS-KEY ((((injur*) AND (prevent*) AND (program*)) OR ((injur*) AND (prevent*)) OR program*) OR ("warm up" OR warm-up) OR (neuromuscular OR "neuromuscular training" OR "integrative neuromuscular training" OR nmt))) AND (TITLE-ABS-KEY (("athletic performance" OR "neuromuscular performance" OR performance) OR (balance OR "dynamic balance" OR "static balance" OR stability OR "postural balance") OR (biomechanic* OR kinetic* OR kinematic*))) AND (TITLE-ABS-KEY ((jump*) OR (land*) OR (run*) OR (hop*) OR (cut*) OR (locomot*) OR ("motor skill" OR "movement skill" OR skill*)))
Web of Science	TOPIC: (child* OR kid* OR adolescent* OR youth OR young) AND TOPIC: (((injur* AND prevent*) OR (injur* AND prevent* AND program*) OR program*) OR ("warm up" OR warm-up) OR (neuromuscular OR "neuromuscular training" OR "integrative neuromuscular training" OR NMT)) AND TOPIC: (("athletic performance" OR "neuromuscular performance" OR performance) OR (balance OR "dynamic balance" OR "static balance" OR stability OR "postural balance") OR (biomechanic* OR kinetic* OR kinematic*)) AND TOPIC: (jump* OR land* OR run* OR hop* OR cut* OR locomot* OR ("motor skill" OR "movement skill" OR skill*))
SPORTSDiscus	(child* OR kid* OR adolescent* OR youth OR young) AND (((injur* AND prevent* AND program*) OR (injur* AND prevent*) OR program*) OR ("warm up" OR warm-up) OR (neuromuscular OR "neuromuscular training" OR "integrative neuromuscular training" OR NMT)) AND (("athletic performance" OR "neuromuscular performance" OR performance) OR (balance OR "dynamic balance" OR "static balance" OR stability OR "postural balance") OR (biomechanic* OR kinetic* OR kinematic*)) AND (jump* OR land* OR run* OR hop* OR cut* OR locomot* OR ("motor skill" OR "movement skill" OR skill*))

Appendix B. Risk of bias Assessment

Table B1. Physiotherapy Evidence Database (PEDro) scores.

Study	Eligibility Criteria Specified⁺	Random Allocation	Concealed Allocation	Baseline Comparability	Blinded Subjects	Blinded Therapists	Blinded Assessors	Adequate Follow-Up	Intention-to-Treat Analysis	Between Group Comparisons	Point Estimates and Variability	Total Score
Kilding et al., 2008	0	1	0	1	0	0	0	1	1	1	1	6/10
Steffen et al., 2008	1	1	0	1	0	0	0	1	1	1	1	6/10
Lim et al., 2009	0	1	0	1	0	0	0	1	1	1	1	6/10
DiStefano et al., 2010	1	1	0	1	0	0	0	1	1	1	1	6/10
Vescovi & VanHeest, 2010	1	1	0	1	0	0	0	0	1	1	1	5/10
Reis et al., 2013	0	1	0	0	0	0	0	1	1	1	1	5/10
Brown et al., 2014	1	1	0	1	0	0	0	0	1	1	1	5/10
Zech et al., 2014	1	1	1	1	0	0	0	1	1	1	1	7/10
Root et al., 2015	1	1	0	1	1	1	1	1	0	1	1	8/10
Rössler et al., 2016	1	1	1	1	0	0	0	1	1	1	1	7/10
Ayala et al., 2017	1	1	1	1	0	0	1	0	1	1	1	7/10
Ondra et al., 2017	1	1	0	1	0	0	0	1	1	1	1	6/10
Akbari et al., 2018 and 2019	1	1	0	1	0	0	0	1	1	1	1	6/10
De Ste. Croix et al., 2018	0	1	1	1	0	0	0	1	1	1	1	7/10
Gatterer et al., 2018	0	1	0	1	0	0	0	1	1	1	1	6/10
Pomares-Noguera et al., 2018	1	1	1	1	0	0	0	1	1	1	1	7/10
Taylor et al., 2018	1	1	1	1	0	0	0	1	1	1	1	7/10
Zarei et al., 2018	0	1	0	1	0	0	0	0	1	1	1	5/10
Zarei et al., 2018	1	1	0	1	0	0	0	0	1	1	0	4/10
McKenzie et al., 2019	0	1	0	1	0	0	0	0	1	1	1	5/10
Pardos-Mainier et al., 2019	0	1	0	0	0	0	0	1	1	1	1	5/10
Parsons et al., 2019	1	1	0	1	0	0	1	1	1	1	1	7/10
Trajkovic & Bogataj, 2020	1	1	0	1	0	0	0	1	1	1	1	6/10

Forrest et al., 2020	1	1	1	1	0	0	0	1	1	1	1	7/10
Font-Lladó et al., 2020	1	1	0	1	0	0	1	1	0	1	1	6/10
Teixeira et al., 2021	1	1	0	1	1	0	0	0	0	1	1	5/10

⁺ Not included in the total score.

Appendix C. Risk of publication bias plots

Figure C1. Funnel plot for vertical jump.

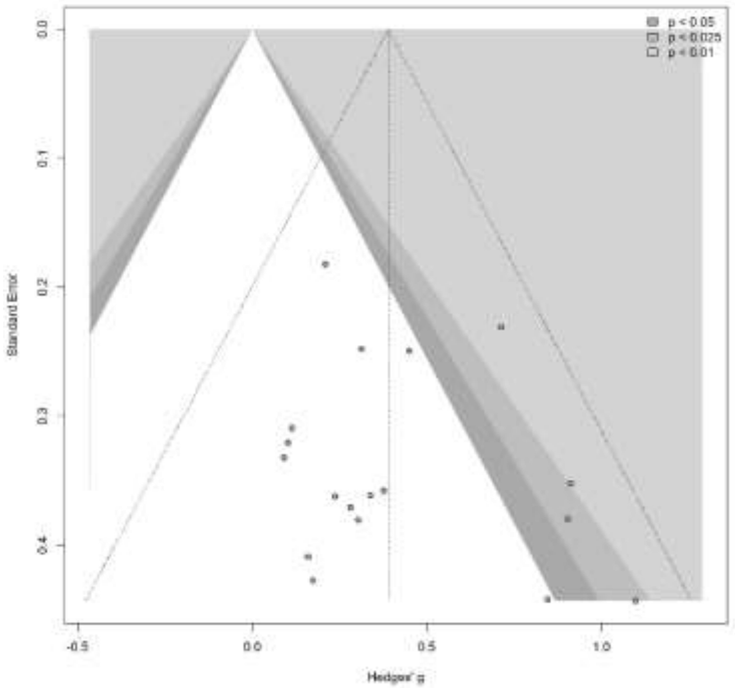

Figure C2. Funnel plot for horizontal jump.

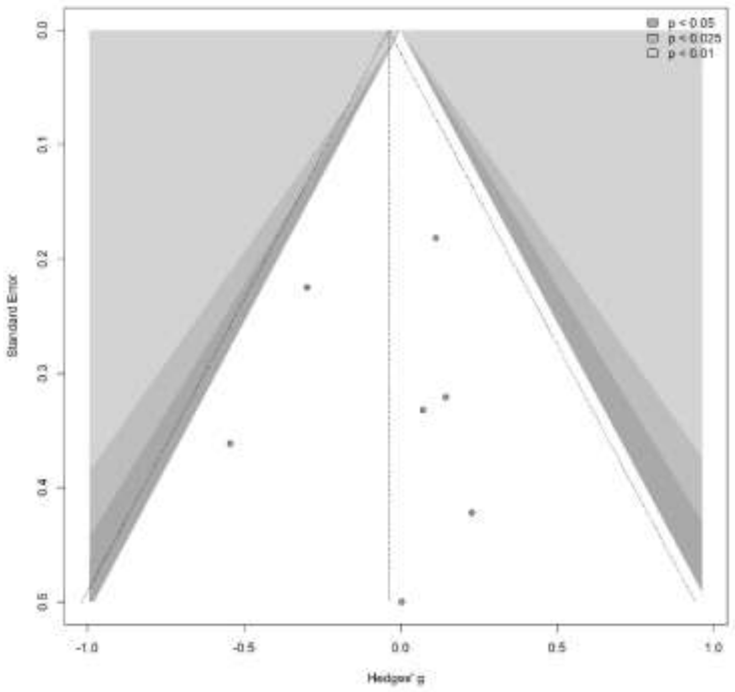

Figure C3. Funnel plot for dynamic balance.

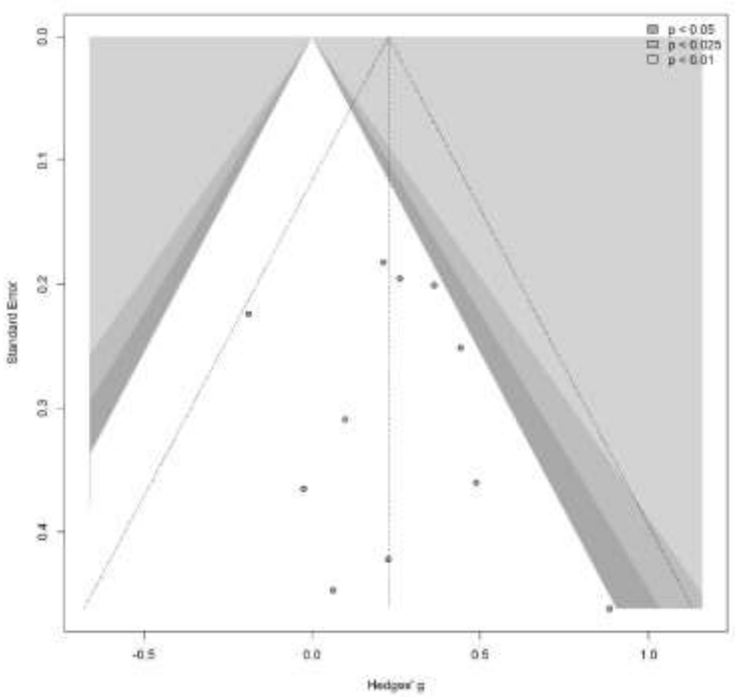

Figure C4. Funnel plot for running speed.

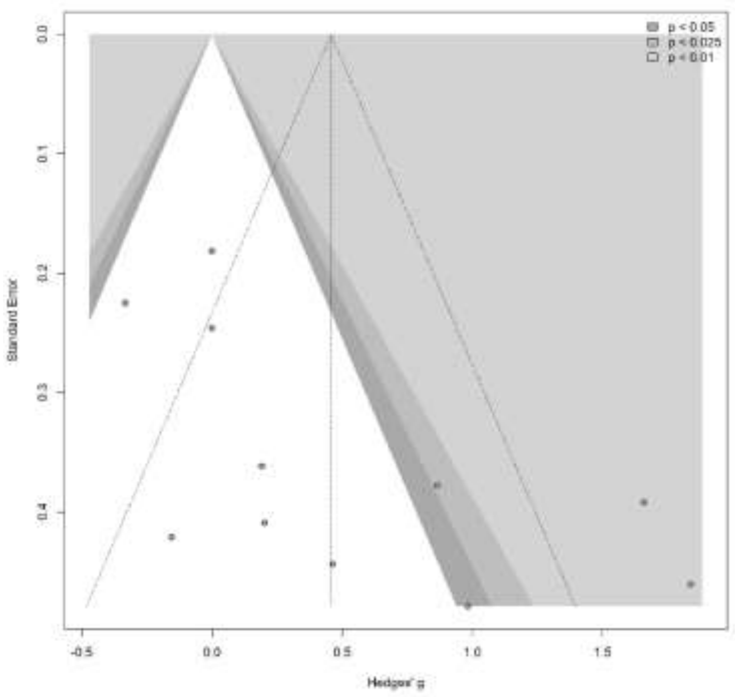

Figure C5. Funnel plot for running acceleration.

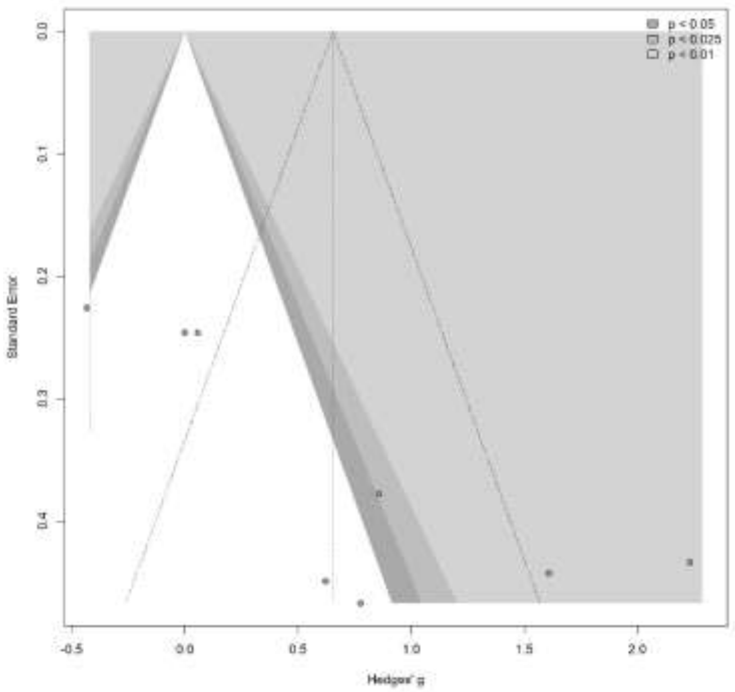

Figure C6. Funnel plot for dribbling.

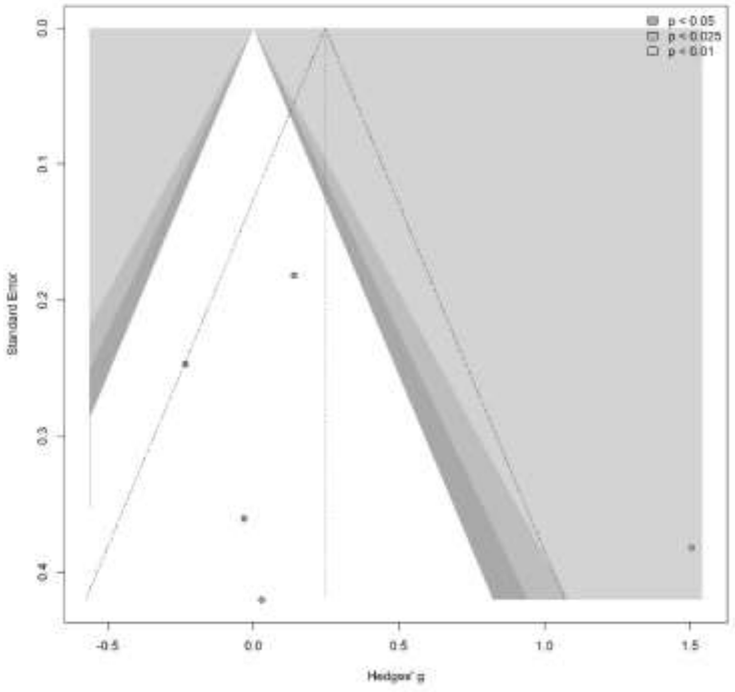

Supplement: Supplemental Material - Effects of Multicomponent Injury Prevention Programs on Children and Adolescents’ Fundamental Movement Skills: A Systematic Review With Meta-Analyses [file sj-pdf-1-ahp-10.1177_08901171221146434.pdf]
